# The Biological Roles and Molecular Mechanisms of Long Non-Coding RNA MEG3 in the Hallmarks of Cancer

**DOI:** 10.3390/cancers14246032

**Published:** 2022-12-07

**Authors:** Lei Zhang, Fuqiang Zhao, Wenfang Li, Guanbin Song, Vivi Kasim, Shourong Wu

**Affiliations:** 1 Key Laboratory of Biorheological Science and Technology, Ministry of Education, College of Bioengineering, Chongqing University, Chongqing 400044, China; 2 The 111 Project Laboratory of Biomechanics and Tissue Repair, College of Bioengineering, Chongqing University, Chongqing 400044, China; 3 Chongqing Key Laboratory of Translational Research for Cancer Metastasis and Individualized Treatment, Chongqing University Cancer Hospital, Chongqing University, Chongqing 400030, China

**Keywords:** maternally expressed gene 3 (MEG3), competing endogenous RNA, long non-coding RNA, microRNA, hallmarks of cancer

## Abstract

**Simple Summary:**

MEG3 is a class of lncRNA, which is considered a tumor suppressor. It is lost or decreased in different biological processes of various human tumors and is closely related to various diseases. MEG3 can modulate the expression of target genes through transcription, translation, post-translational modification and epigenetic regulation. Studies have shown that MEG3 dysfunction has been linked to a poor prognosis and drug resistance. MEG3 mediates the hallmarks of cancer through a variety of mechanisms, acting as a tumor suppressor to limit tumor growth. Hence, MEG3 is a potential prognostic marker and antitumor therapeutic target.

**Abstract:**

Long non-coding RNAs (lncRNAs) are critical regulators in various biological processes involved in the hallmarks of cancer. Maternally expressed gene 3 (MEG3) is lncRNA that regulates target genes through transcription, translation, post-translational modification, and epigenetic regulation. MEG3 has been known as a tumor suppressor, and its downregulation could be found in various cancers. Furthermore, clinical studies revealed that impaired MEG3 expression is associated with poor prognosis and drug resistance. MEG3 exerts its tumor suppressive effect by suppressing various cancer hallmarks and preventing cells from acquiring cancer-specific characteristics; as it could suppress tumor cells proliferation, invasion, metastasis, and angiogenesis; it also could promote tumor cell death and regulate tumor cell metabolic reprogramming. Hence, MEG3 is a potential prognostic marker, and overexpressing MEG3 might become a potential antitumor therapeutic strategy. Herein, we summarize recent knowledge regarding the role of MEG3 in regulating tumor hallmarks as well as the underlying molecular mechanisms. Furthermore, we also discuss the clinical importance of MEG3, as well as their potential in tumor prognosis and antitumor therapeutic strategies.

## 1. Introduction

Cancer is the main cause of death globally and a significant impediment to extending life expectancy. In 2020, there was an estimated 19.3 million new cases of cancer and nearly 10 million cancer-related mortality globally [1]. While cancers that are accessible for early identification are slowing down, other prevalent malignancies are making significant progress [2]. Hence, there is an urgent need to find novel prognostic biomarkers and tumor therapeutic targets to combat cancer. 

Tumorigenesis as well as the malignant transformation from benign tumors to malignant cancers is a complex process due to aberrant gene expressions. Distinct from normal cells, tumor cells have gained special characteristics, which are known as “hallmarks of cancer”, including sustaining proliferative signaling, resisting cell death, inducing angiogenesis, activating invasion and metastasis, and metabolic reprogramming [3,4]. Besides mutations in protooncogenes and tumor suppressor genes, impaired gene expression regulatory pathways such as transcriptional, translational, post-translational, or epigenetic regulations are also the main reasons for tumorigenesis and malignant transformation [5,6]. In the last two decades, numerous studies have revealed that non-coding RNA (ncRNA), such as microRNA (miRNA), long non-coding RNA (lncRNA), circular RNA (circRNA), small interfering RNA (siRNA), and RNA interacting with piwi proteins (piRNA), are crucial regulators of gene expression. These ncRNAs could exert their regulatory functions by regulating various steps of gene expression, that is, transcription, post-transcriptional modifications, translation, post-translational modifications, chromatin remodeling, and signal transduction [7,8].

Long non-coding RNAs (lncRNAs) are a class of non-coding RNAs more than 200 nucleotides in length. LncRNAs can act as competing endogenous RNA (ceRNA) that sponges and blocks the effect of miRNAs, a class of ncRNA that suppresses target genes expression at their translational level by binding to their 3′ untranslated regions (3′ UTR) [9]. Furthermore, lncRNAs could interact with DNA, RNA, protein molecules and/or their complexes, acting as an essential regulator in transcriptional, post-transcriptional, and chromatin remodeling regulations [10]. Interestingly, recent studies found that some lncRNAs contain short open reading frames (sORFs), which can encode small proteins or micropeptides to exert their physiological roles [11,12]. Aberrant lncRNA expression, which could be caused by single nucleotide polymorphism (SNPs), copy number alterations, and mutations, has been found in various tumors such as colorectal cancer (CRC), thyroid cancer, and ovarian cancer (OC), and is closely related with cancer hallmarks and malignant transformation [13,14,15]. Thus, lncRNAs have gained attention as novel biomarkers for tumors and as targets for antitumor therapeutic strategies [16].

**Figure 1:**
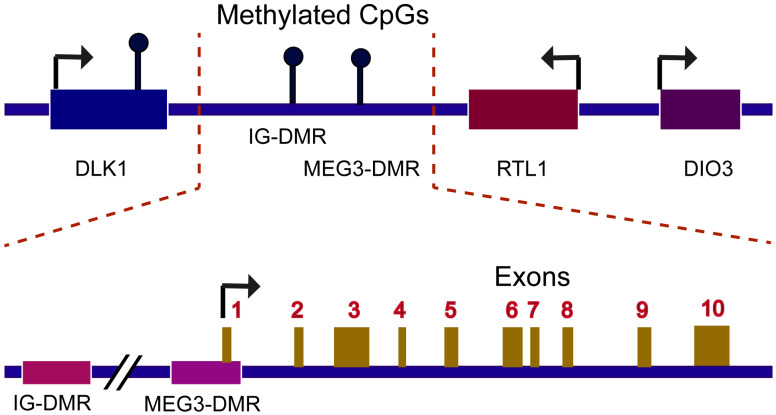
Schematic diagram of DLK1-MEG3 locus on human chromosome 14. The 837 kb-long DLK1-MEG3 locus contains the protein-coding genes DIO3, RTL1, and DLK1. The *MEG3* gene has ten exons and is 35 kb long. The IG-DMR is 13 kb upstream of the MEG3 gene. The MEG3-DMR overlaps with the MEG3 promoter. IG-DMR: intergenic differentially methylated region.

Maternally expressed gene 3 (MEG3) is an imprinted gene with an approximate length of 35 kbp and found at the DLK1-MEG3 locus on human chromosome 14q32.3 [17]. Its mouse homolog, *gene trap loci 2* (*Gtl2*), is located on mouse distal chromosome 12 [18]. MEG3 is transcribed by RNA polymerase 2 and spliced into 10 exons containing five key structural motifs (M-I to M-V) [17,19,20,21]. Mature MEG3 RNA, which is 1.6 kbp in length, is polyadenylated at its 3′ ends, and is located both in the nucleus and cytoplasm [22,23] (Figure 1). MEG3 could impact various diseases, including ischemic neuronal death, atherosclerosis and type 2 diabetes mellitus [24,25,26]. Recent studies revealed that MEG3 expression decreased in a wide variety of tumors, playing a crucial role as a tumor suppressor [27,28]. The first study regarding the role of MEG3 in tumors was reported by Zhang et al. They found the defect of MEG3 expression in pituitary adenomas, and that ectopic expression of this gene suppressed tumor cell growth [29]. More recently, Moradi et al. reported that MEG3 could function as a ceRNA that interact directly with multiple genes or proteins, including p53, enhancer of zeste homologue 2 (EZH2), and nuclear factor-kappa B (NF-κB). MEG3 exerts its tumor-suppressive effect by regulating various cancer hallmarks, as it could inhibit tumor cell proliferation, induce cell death, reduce invasion and metastasis, prevent angiogenesis, and inhibit tumor cells’ metabolic reprogramming. 

In this review, we summarized the current knowledge regarding the expression level, functions, as well as the molecular mechanism underlying MEG3 regulation on cancer hallmarks. Furthermore, we highlighted the clinical significance of MEG3 as a biomarker for cancer prognosis, as well as a novel therapeutic strategy for cancer therapy.

## 2. Mechanism of MEG3 Regulations

### 2.1. MEG3 Could Sponge miRNAs

LncRNAs can function as ceRNAs that bind to target miRNAs like a sponge and prevent miRNA from binding to its target mRNA, thus affecting the mRNA abundance of the target gene and their protein levels [30,31]. Numerous studies have reported that MEG3 can function as ceRNA by sponging and sequestering miRNAs, such as miR-21, miR-181a, and miR-421, from their target genes [32,33,34,35,36,37,38,39,40,41,42]. Similar to the targets of the miRNAs it regulates, MEG3 possesses microRNA response elements (MREs). Through these MREs, MEG3 binds to the miRNA binding sites competitively with the corresponding target mRNAs, thereby removing the target mRNAs and eliminating the inhibitory effect of miRNA on them. The lncRNA-miRNA-mRNA forms a complex network of action, whose homeostasis is crucial for maintaining normal physiological conditions. Meanwhile, disruption of this homeostasis is closely related to diseases including cancers [43]. 

### 2.2. MEG Regulations on Target Genes Transcription

**Figure 2:**
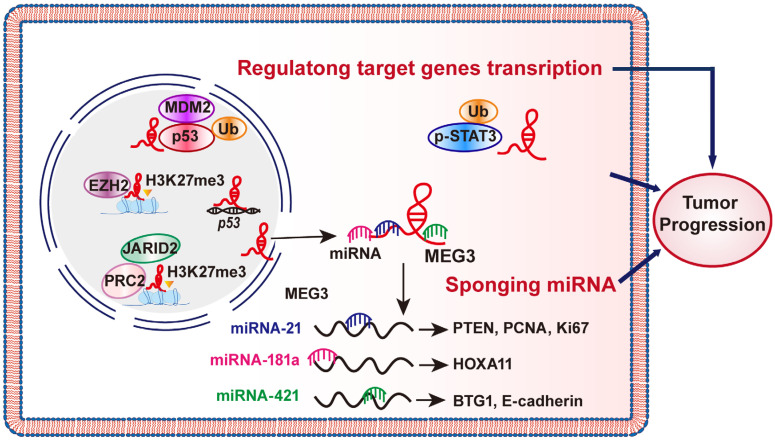
MEG3 inhibits cancer progression through different mechanisms. MEG3 is involved in tumor progression in two ways, such as acting as a sponge for miRNA and regulating its targets through transcriptional as well as post-translational regulations.

Besides as a ceRNA, MEG3 can regulate its targets through transcriptional as well as post-translational regulations (Figure 2). For example, MEG3 could promote p53 expression by promoting its transcriptional activity and post-translational modification. MEG3 could enhance p53 transcriptional activity, thereby increasing p53 expression level and negatively regulating the cell cycle [44,45] Furthermore, MEG3 could also decrease the level of murine double minute 2 (MDM2), an E3 ubiquitin ligase that enhances p53 ubiquitination/proteasomal degradation, leading to p53 protein stabilization and transcriptional activation of p53 downstream targets [46]. Meanwhile, Weng et al. also found that MEG3, through its 732-1174 nucleic acid region, binds directly to Clusterin (CLU) protein and impedes CLU’s interactions with its target proteins, such as vascular endothelial growth factor (VEGF) or matrix metalloproteinase (MMP-9) [47]. MEG3 affects the stability of proteins by regulating their post-translational modifications. Zhang et al. showed that MEG3 could suppress the accumulation of the phosphorylated signal transducer and activator of transcription 3 (p-STAT3) protein by recruiting ubiquitination enzymes and thus directing pSTAT3 into ubiquitin/proteasomal degradation pathway without affecting its phosphorylation. This in turn suppresses the p-STAT3/c-Myc axis, and subsequently, leads to a decrease in cell proliferation potential [48]. 

Several studies have reported that lncRNAs are involved in chromatin remodeling by directing the recruitment of chromatin modifiers to target gene sites, for example, by associating with polycomb repressive complex 2 (PRC2) and inducing the trimethylation of histone H3 lysine 27 (H3K27me3) [49,50]. MEG3 could function as a molecular scaffold linking different proteins and forming large complexes that regulate chromatin structure and gene expression. By interacting with the RNA binding domain of Jumonji and AT-rich interaction domain containing 2 (JARID2), MEG3 stimulates PRC2 and JARID2 assembly, thereby enhancing H3K27me3 recruitment and suppressing the transcription of E-cadherin and miR-200 family [51].

MEG3 could also induce H3K27me3 by interacting with EZH2, a catalytic subunit of the PRC2 complex. Through this regulation, MEG3 induces the deposition of H3K27me3 in the distal regulatory region (DRE) of the transforming growth factor-β (TGF-β) gene, thereby inhibiting TGF-β gene transcription in trans, and subsequently, the transcription of TGF-β pathway genes transforming growth factor beta receptor 1 (TGFBR1), transforming growth factor beta 1 (TGFB1), and SMAD family member 2 (SMAD2) [52]. Similarly, MEG3 inhibits engrailed-2 (EN-2) expression by EZH2-mediated H3K27me3 [53]. Interestingly, MEG3 could also interact with EZH2 protein and stimulates its ubiquitination/proteasomal degradation; EZH2 could in turn suppress MEG3 through its interaction with DNA methyltransferase 1 (DNMT1) and histone deacetylase 1 (HDAC1), thereby suppressing MEG3 transcription by inducing DNA methylation. Hence, the regulation of MEG3 on EZH2 forms a concerted negative feedback loop [54].

## 3. MEG3 Regulates Various Hallmarks of Cancer

Recent studies revealed that MEG3 is associated with hallmarks of cancer, including proliferation, cell death, invasion and metastasis, metabolic reprogramming, and angiogenesis, by regulating various pathways (Table 1). MEG3 inhibits cancer progression through different mechanisms. MEG3 is involved in tumor progression in two ways, such as acting as a sponge for miRNA and regulating its targets through transcriptional as well as post-translational regulations. For example, MEG3 is closely related to the expression level of p53, a tumor suppressor whose mutation could be found in more than 50% of cancer patients [55]. MEG3 can directly interact with the DNA binding domain of p53 thereby enhancing the transcription of numerous p53 target genes [56]. MEG3 can also regulate p53 expression level indirectly by decreasing MDM2 protein level, leading to the decrease in MDM2-mediated p53 ubiquitination/proteasomal degradation, thereby stabilizing p53 protein levels [57,58]. 

### 3.1. MEG3 Inhibits Tumor Cell Proliferation

Abnormal, uncontrolled cell growth due to the dysregulation of cell proliferation is the most fundamental cause of tumorigenesis. Aberrant MEG3 expression has been observed in various tumors and is closely linked with tumor cell proliferation [76]. MEG3 could up-regulate OTU deubiquitinase 4 (OTUD4) and RNA binding motif single-stranded interacting protein 3 (RBMS3) by sponging miR-494 and miR-141-3p, respectively, thereby suppressing breast cancer cells proliferation [59,77]. MEG3 could inhibit the growth and proliferation of T-cell lymphoblastic lymphoma by sponging miR-214, thereby activating apoptosis-inducing factor mitochondrion-associated 2 (AIFM2) expression [61]. By sponging miR-494 and miR-374a-5p, MEG3 can up-regulate phosphatase and tensin homolog (PTEN), resulting in cell growth inhibition in bladder cancer and pancreatic ductal adenocarcinoma [78,79]. MEG3 also can inhibit cholangiocarcinoma proliferation and invasion by inhibiting the major components of the PRC1 complex, B lymphoma Mo-MLV insertion region 1 (Bmi1), and RING finger protein 2 (RNF2) [80].

The cell cycle is an important process that regulates cell proliferation. MEG3 could induce cell-cycle arrest in G_0_/G_1_ phase, thereby suppressing cell proliferation and ultimately inducing cell apoptosis. MEG3 could induce G_0_/G_1_ cell cycle arrest in glioma and ovarian cancer cells by inactivating the Wnt/β-catenin signaling pathway and upregulating PTEN expression, respectively [60,81]. Furthermore, by sponging its target miRNAs, such as miR-10a-5p, MEG3 can cause G0/G1 cell cycle arrest and enhance the expression of PTEN, Bcl-2-associated X (Bax), and p53 protein in hepatocellular carcinoma (HCC) [82], or by sponging miR-7 and miR-376, leading to the downregulation of miR-7/RAS like family 11 member B (RASL11B) and miR-376/protein kinase D1 (PKD1) axis [62,63]. 

MEG3 could also inactivate the PI3K/Akt and ERK pathways, which are crucial for cell proliferation [3]. In renal cell carcinoma, MEG3 upregulates β-galactoside α-2,3-sialyltransferase 1 (ST3Gal1) through its interaction with transcription factor c-Jun, leading to the decrease in epithelial growth factor receptor (EGFR) phosphorylation and PI3K/Akt pathway inactivation [83]. Meanwhile, in gliomas and hemangiomas, MEG3 could inactivate PI3K/Akt pathway by sponging miR-93 and miR-494, respectively [84,85]; in pancreatic neuroendocrine tumors, MEG3 downregulates brain protein I3 (BRI3) expression by sponging miR-183, leading to the inactivation of p38/ERK/Akt and Wnt/β-catenin signaling pathways [64]. 

Cancer stem cells (CSCs) are a small population of tumor cells that are usually in the dormant stage and have been assumed to be the main reason for tumorigenesis potential, tumor metastasis, recurrence, and drug resistance [86]. Targeting CSCs has been considered a potential therapeutic strategy for eradicating cancers; however, they are significantly less sensitive to current chemotherapy- and radiotherapy-based antitumor therapeutic strategies, as these strategies target proliferative cells [87]. MEG3 can repress CSC self-renewal ability and decrease cancer stemness phenotype in oral CSCs by blocking miR-421 [88]. Furthermore, by sponging miR-708, MEG3 enhances SOCS3 expression, thereby decreasing colorectal CSCs stemness by suppressing STAT3 signaling [89].

### 3.2. MEG3 Induces Cell Death

Apoptosis is a programmed cell death controlled by a signaling cascade to maintain a stable internal environment. The elimination of cancer cells by apoptosis has been a key cue in clinical cancer treatment [90]. Apoptosis could be divided into intrinsic and extrinsic apoptotic pathways. The intrinsic apoptotic pathway, also known as mitochondria-mediated apoptosis, is regulated by pro-apoptotic B-cell lymphoma 2 (Bcl-2) proteins, anti-apoptotic Bcl-2 proteins, and BH3-only proteins, which triggers the activation of executor caspases 3 and 7 by activating caspase 8. Meanwhile, the extrinsic apoptotic pathway is regulated by death receptors, such as the tumor necrosis factor (TNF) receptor, which promotes the cleavage of initiator caspase, caspase 9, subsequently activating executor caspases [91,92]. 

Previous reports have shown that MEG3 could enhance intrinsic apoptosis by various mechanisms. In prostate cancer, osteosarcoma, urinary tract epithelial cancer and pituitary tumor cells, MEG3 could directly bind to miR-361-5p, miR-96, and miR-376B-3p, leading to the promotion of forkhead box M1 (FoxM1) and tropomyosin 1 (TPM1) expression while repressing oncogene high mobility group AT-hook 2 (HMGA2) expression. This results in the reduction in Bcl-2 and the rising of Bax protein levels, as well as the increase in caspases-3 and -9 cleavages, thereby inducing tumor cell apoptosis [93,94,95,96]. In oral squamous cell carcinoma (OSCC) and CML, MEG3 promoted apoptosis by sponging miR-548d-3p and miR-147, thereby promoting suppressor of cytokine signaling 5 (SOCS5) and suppressor of cytokine signaling 6 (SOCS6) expression while inhibiting the JAK-STAT signaling pathway [65,66].

MEG3 could also trigger apoptosis in ESCC and CRC by increasing endoplasmic reticulum (ER) stress-related proteins, including glucose-regulated protein 78 (GRP78), activating transcription factor 6 (ATF6), protein kinase R-like endoplasmic reticulum kinase (PERK), and C/EBP-homologous protein (CHOP), leading to enhanced caspases-9 and -3 cleavages [97,98]. Furthermore, MEG3 could activate apoptotic cascade in laryngeal cancer by sponging miR-23a and promotes apoptotic protease activating factor-1 (APAF-1) expression [99], and in gallbladder cancer by promoting EZH2 ubiquitination/proteasomal degradation. This in turn suppressed the expression level of its downstream target tumor suppressor large tumor suppressor 2 (LATS2), thus increasing the levels of cleaved PARP, Bax, and Bcl-2 [100]. Moreover, it could also downregulate miR-21-5p, leading to an increase in p53 and caspase 3 cleavage protein cleavage [67].

Besides intrinsic apoptosis, MEG3 could also trigger extrinsic apoptosis pathways. In cholangiocarcinoma and gallbladder cancer, MEG3 stimulated NF-κB signaling pathway and triggered apoptosis by sponging miR-361-5p expression and activating TNF receptor-associated factor 3 (TRAF3) [101,102]. 

Autophagy is an intracellular self-destructive form of cell death that transfers cytoplasmic proteins or organelles to the lysosome to fulfill the metabolic and self-renewal needs of organelles and the cell itself [103,104,105]. Previous studies have reported that MEG3 could attenuate autophagy by suppressing the forkhead box O1 (FOXO1) expression, leading to the decrease in autophagy-related proteins microtubule-associated protein light chain 3 II (LC3 II), beclin 1, autophagy related 3 (ATG3), autophagy related 5 (ATG5), and autophagy related 12 (ATG12), as well as the increase in the autophagy substrate p62 [106]. Hence, MEG3 regulation on autophagy needs further investigation.

### 3.3. MEG3 Negatively Regulates Tumor Cells Invasion and Metastasis Potentials

Metastasis is a complex process that includes epithelial-mesenchymal transition (EMT), invasion, intravasation, cell survival in circulation, extravasation, and metastatic colonization [107,108]. EMT is the first, initiative event in cancer metastasis in which epithelial cells gained mesenchymal characteristics such as decreased intercellular adhesion and increased motility, while losing epithelial characteristics [109,110]. MEG3 could suppress GC cells’ EMT and metastasis potential by sponging miR-21, leading to the increase in the expression of epithelial marker E-cadherin, and a decrease in mesenchymal markers such as N-cadherin, Snail, and β-catenin as well as cell migration markers such as matrix metalloproteinase-2 (MMP-2), matrix metalloproteinase-3 (MMP-3), and MMP-9 [34,111] and by inhibiting the binding between miR-665 and its target, cytokine signaling 3 (SOCS3), thereby enhancing SOCS3 expression and suppressing FAK/Src pathway [112]. Meanwhile, by sponging miR-216a, MEG3 enhances programmed death-1 (PD-1) expression while suppressing EMT inducer myeloid cell leukemia-1 (MCL-1) in endometrial cancer cells [113]. In OC cells, MEG3 could inhibit tumor cells migration and invasion potentials by sponging up miR-219a-5p and miR-30e-3p, resulting in the downregulation of EGFR and increase in laminin subunit alpha 4 (LAMA4), respectively [68,114]. Meanwhile, by sponging miR-19a, MEG3 enhanced PTEN expression, thereby suppressing glioma cell migration and invasion potentials [69]. Furthermore, in HCC, MEG3 could inhibit metastasis by sponging miR-544b and miR-5195-3p, thereby upregulating target genes B-cell translocation gene (BTG2) and FOXO1 expression [70,115]. Moreover, MEG3 could also suppress EMT by blocking the phosphoserine aminotransferase 1 (PSAT1)-dependent glycogen synthase kinase (GSK)-3β/Snail signaling [116].

The link between MEG3 and metastasis has also been confirmed by clinical samples from thyroid cancer (TC) patients showing that MEG3 downregulation was associated with lymph node metastasis. MEG3 could suppress TC cell migration and invasion by downregulating *Rac family small GTPase 1* (*Rac1*) expression by targeting its 3′ UTR [117]. Furthermore, MEG3 competitively interacts with miR-27a as the ceRNA of PH domain and leucine-rich repeat protein phosphatase 2 (PHLPP2) mRNA, promoting PHLPP2 protein translation and inhibiting c-Jun phosphorylation and c-Jun-mediated *c-Myc* mRNA transcription, thereby impairing invasion and lung metastasis of bladder cancer cells [71]. 

### 3.4. MEG3 Regulation on Tumor Cells Metabolic Reprogramming

Metabolic alteration is a characteristic of tumor cells crucial for supporting their rapid cell growth [3]. Unlike normal cells, which mainly depend on glycolysis followed by oxidative phosphorylation, tumor cells prefer inefficient aerobic glycolysis with a significantly higher turnover rate compared to normal cells even under adequate oxygen availability. This phenomenon is known as the Warburg effect [118,119]. The reprogrammed metabolic network generates intermediates, such as those involved in the glycolysis or tricarboxylic acid (TCA) cycle processes, which benefit cancer cells by helping them meet their energy needs as well as anabolic and redox and building blocks demands in the early stages of cancer development [120]. MEG3 activated by vitamin D can inhibit aerobic glycolysis and lactic acid production in CRC cells by inducing ubiquitin-dependent c-Myc degradation, thereby inhibiting c-Myc target genes expression involved in the glycolysis pathway, such as lactate dehydrogenase A (LDHA), pyruvate kinase muscle 2 (PKM2) and hexokinase 2 (HK2) [72]. Furthermore, MEG3 can promote succinate dehydrogenase (SDH) expression by sponging miR-361-5p, leading to an increase in succinate, a key TCA metabolite, thereby suppressing OSCC progression [73]. 

### 3.5. MEG3 Suppresses Tumor Angiogenesis

Formation of new blood vessels in tumor tissues from existing blood vessels is crucial for supplying tumor cells with oxygen and nutrient, for adapting to the fluctuating oxygen pressure in their microenvironment, as well as for metastasis [121]. This process involved many angiogenic factors, including vascular endothelial growth factor A (VEGFA), basic fibroblast growth factor (bFGF), and angiogenin. These factors increase endothelial cell development and vascular permeability, resulting in the formation of new blood vessels [122]. The role of MEG3 in tumor angiogenesis remains intriguing. Zhang et al. reported that MEG3 can suppress angiogenesis-related gene VEGFA, placental growth factor (PGF), bFGF, transforming growth factor β1 (TGF-β1) and MMP-9 expression by decreasing phosphorylated levels of AKT and inhibiting AKT pathway, ultimately suppressing angiogenesis in breast cancer [74]. However, Li et al. demonstrated that MEG3 could promote angiogenesis in lung carcinoma, as it could significantly increase the expression of angiogenesis-related factors VEGFA, vascular endothelial growth factor B (VEGFB), bFGF, stromal cell-derived factor-1 (SDF-1), transforming growth factor β (TGF-β), angiogenin, and MMP-9 [75]. The reasons underlying this discrepancy need further investigation.

## 4. Clinical Significance of lncRNA MEG3

### 4.1. MEG3 Is a Potential Biomarker for Tumor Prognosis

Decreased expression of MEG3 was associated with poor prognosis in a variety of human malignancies [123]. As shown in Table 2, MEG3 has been proven to have anti-tumor effects, and potential prognostic and clinical significance in various human cancers [60,76,123,124,125,126,127,128,129,130]. 

Analysis of MEG3 expression in glioma patients showed that low expression of MEG3 was associated with poor overall survival rates, advanced WHO grade, low Karnofsky performance score (KPS), isocitrate dehydrogenase (IDH) wild-type, and tumor recurrence [60,125]. Xu et al. revealed that the copy number variation (CNV) levels of MEG3 were positively associated with overall survival and progression-free survival compared to the wild-type in low-grade glioma [123]; Gao et al. revealed, using 63 patients with retinoblastoma, that hypermethylation of *MEG3* promoter was highly associated with poor survival, further confirming that MEG3 expression level is negatively correlated with poor prognosis [128]. Meanwhile, using 58 clinical ESCC tissues, Ma et al. found that low MEG3 expression was correlated with tumor size, lymph node metastasis, clinical stage, and poor prognosis [126]. These results were in accordance with other studies involving 48 CRC cases [129]. Furthermore, a negative correlation between MEG3 expression and short overall survival, relapse-free survival, and poor prognosis has also been found in breast cancer, NSCLC, and glioblastoma [76,127,130]. Together, these results show a negative correlation between MEG3 and tumor progression as well as prognosis, indicating the potential of using MEG3 as a biomarker for tumor prognosis. 

### 4.2. MEG3 Is a Potential Target for Tumor Therapy

Anti-tumor therapies have been evolving and improving in recent years, yet resistance to chemotherapy, radiotherapy, targeted therapy, and immunotherapy remains a major problem [131]. Cytotoxic anti-tumor drugs such as cisplatin, paclitaxel, and doxorubicin, as well as targeted medicines such as imatinib, have been used for clinical cancer treatment. However, the persistent rise of drug resistance seriously undermines their efficacies [132]. MEG3 can facilitate chemotherapeutic drug sensitivity and radiosensitivity by altering key signaling pathways, making it a novel therapeutic strategy for cancer treatment (Table 3). 

Assessment using 90 peritoneal biopsies of high-grade serous OC showed that MEG3 expression is associated with sensitivity to platinum-based chemotherapy [133]. MEG3 can act as an agonist of cisplatin in suppressing triple-negative breast cancer (TNBC) growth and metastasis potentials, and facilitate pyroptosis by activating cisplatin-induced NLRP3/caspase-1/gasdermin D (GSDMD) pathway [134]. MEG3 can also enhance NSCLC sensitivity to cisplatin by sponging miR-21-5p and thereby upregulating SRY-box transcription factor 7 (SOX7) expression [135]; by sponging miR-141, MEG3 could overcome CRC cells chemoresistance to oxaliplatin and promote programmed cell death factor 4 (PDCD4) expression [129]. Subsequently, MEG3 could suppress cisplatin and cyclophosphamide resistance in T-cell lymphoblastic lymphoma cells through the PI3K/mTOR pathway [136]. 

MEG3 could also act as an agonist of other antitumor drugs. Through MEG3/miR-4513/phenazine biosynthesis-like domain-containing (PBLD) axis, MEG3 promoted breast cancer cells’ sensitivity to paclitaxel [137]. Furthermore, MEG3 suppresses the levels of drug-resistant transporters, including multidrug resistance-associated protein-1 (MRP1), multidrug resistance protein 1 (MDR1), and ATP binding cassette subfamily G member 2 (ABCG2), thus increasing CML cells’ sensitivity against imatinib; miR-21 mimics could reverse their levels [138]. Meanwhile, by sponging miR-155, MEG3 upregulated alpha-1,2-mannosyltransferase (ALG9) expression, thereby promoting AML cells’ sensitivity against adriamycin and vincristine [139]. Moreover, MEG3 could promote pancreatic cancer cells’ chemoresistance to gemcitabine [140].

Besides, MEG3 was closely related to ^131^I-sensitivity of thyroid carcinoma by sponging miR-182 [141]. Finally, very recent research showed that tumor-targeting therapy of osteosarcoma (OS) can be performed by a highly effective engineered and MEG3-loaded exosome, as a combination of MEG3 and exosome significantly increased MEG3 therapeutic effect [142]. Together, these findings suggest that MEG3 plays a significant role in enhancing chemotherapeutic drug sensitivity and radiosensitivity in a variety of human cancers, making it a potential therapeutic target for cancer treatment.

**Table 3:** Roles of MEG3 in therapeutic resistance of cancers.

Cancer Type	Expression	Target	Chemical-/Radioresistance	Refs
TNBC	Downregulated	NLRP3/caspase-1/GSDMD pathway	Cisplatin (DDP)	[134]
NSCLC	Downregulated	miR-21-5p/SOX7	Cisplatin	[135]
T-cell lymphoblastic lymphoma	Downregulated	PI3K/mTOR signaling	Cisplatin and Cyclophosphamide	[136]
CRC	Downregulated	miR-141/PDCD4	Oxaliplatin	[129]
AML	Downregulated	miR-21/MRP1, MDR1, and ABCG2	Imatinib	[138]
Breast cancer	Downregulated	miR-4513/PBLD	Paclitaxel (PTX)	[137]
ACL	Downregulated	miR-155/ALG9	Adriamycin and Vincristine	[139]
Thyroid carcinoma	Downregulated	miR-182	^131^I	[141]

Abbreviations: TNBC: triple-negative breast cancer; ESCC: esophageal squamous cell carcinoma; CRC: colorectal cancer; AML: chronic myeloid leukemia; ACL: acute myeloid leukemia.

## 5. Conclusions and Perspectives

**Figure 3:**
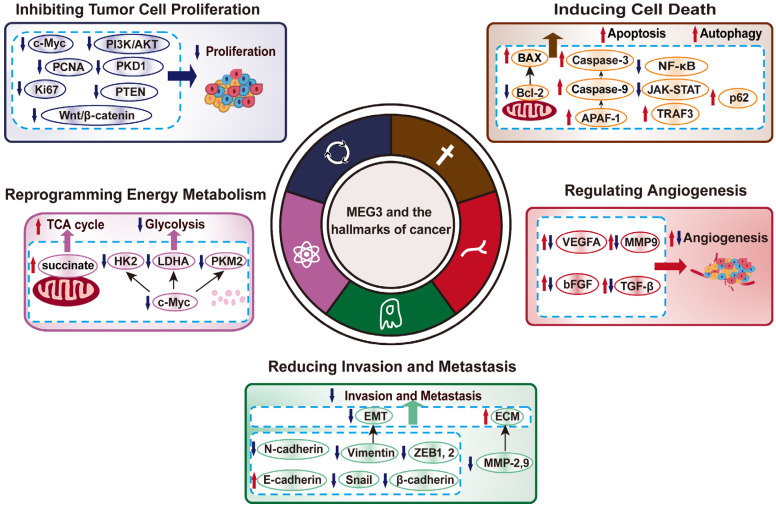
MEG3 and the hallmarks of cancer. In this review, we mainly focus on five hallmarks of cancer regulated by MEG3, including inhibiting tumor cell proliferation, inducing cell death, reducing invasion and metastasis, reprogramming energy metabolism, and regulating angiogenesis.

MEG3 has emerged as a potential tumor suppressor that could regulate various hallmarks of cancer including cell proliferation, cell death, invasion and metastasis, metabolic reprogramming, angiogenesis, and drug resistance (Figure 3). MEG3 expression is downregulated in most malignant tumors, including glioma, HCC, CRC, and breast cancer. As shown in Figure 2, MEG3 regulation on tumor progression occurs through its function as a sponge that adsorbs miRNA, transcription, protein translation and post-translational modifications. However, in some cases, for example in angiogenesis, the role of MEG3 is still unclear, as current studies provide paradoxical results that require further detailed investigation. It is also noteworthy that a recent study showed that MEG3 could promote HCC cell senescence by sponging miR-16-5p, leading to the decrease in vestigial like family member 4 (VGLL4), which is a tumor suppressor and transcriptional cofactor, while increasing the levels of senescence-related markers p21 and p16 [143]. 

Hence, while more detailed studies are still needed to investigate whether MEG3 could regulate other hallmarks of cancer, such as avoiding immune destruction, genome instability and mutation, non-mutational epigenetic reprogramming, unlocking phenotypic plasticity and polymorphic microbiomes and whether there are exceptions for its tumor suppressive effects in certain hallmarks of cancer, present results demonstrate the tumor suppressive function of MEG3. Furthermore, although detailed investigations are still needed, MEG3 is a potential diagnostic biomarker and anti-tumor therapeutic target.

## Figures and Tables

**Table 1:** Biological implications of MEG3 on hallmarks of cancer.

Cancer Type	miRNA	Related Genes	Hallmarks	Refs
Breast cancer	miR-494-3p	OTUD4	Growth inhibition	[59]
Glioma	/	Wnt/β-catenin	Cell cycle regulation	[60]
T-cell lymphoblastic lymphoma	miR-214	AIFM2, Ki-67, PCNA	Growth inhibition	[61]
Clear cell renal cell carcinoma	miR-7	RASL11B	Growth inhibition	[62]
CRC	miR-376	PKD1	Cell cycle regulation	[63]
Pancreatic neuroendocrine tumor	miR-183	BRI3	Growth inhibition	[64]
OSCC	miR-548d-3p	SOCS5, SOCS6	Apoptosis induction	[65]
CML	miR-147	JAK/STAT3	Apoptosis induction	[66]
Cervical cancer	miR-21-5p	p53, caspase3	Apoptosis induction	[67]
Breast cancer	miR-421	E-cadherin	EMT inhibition	[42]
Ovarian cancer	miR-219a-5p	EGFR	EMT inhibition	[68]
Glioma	miR-19a	PTEN	Metastasis inhibition	[69]
HCC	miR-544b	BTG2	Metastasis inhibition	[70]
Bladder cancer	miR-27a	PHLPP2, c-Myc	Metastasis inhibition	[71]
CRC	/	LDHA, PKM2, HK2	Metabolic reprogramming	[72]
OSCC	miR-361-5p	succinate	Metabolic reprogramming	[73]
Breast cancer	/	VEGFA, PGF, bFGF, TGF-β1, MMP-9, AKT	Angiogenesis inhibition	[74]
Lung cancer	/	VEGFA, VEGFB, bFGF, SDF-1, TGF-β, angiogenin, MMP-9	Angiogenesis promotion	[75]

Abbreviations: CRC: colorectal cancer; OSCC: oral squamous cell carcinoma; CML: chronic myeloid leukemia; HCC: hepatocellular carcinoma; EMT: epithelial-mesenchymal transition.

**Table 2:** MEG3 expression and relevant clinical characteristics in human cancers.

Cancer Type	Expression	Relevant Clinical Characteristics	Refs
Glioma	Downregulated	Overall survival rates, Advanced WHO grade, Karnofsky performance score, IDH wild-type, tumor recurrence, progression-free survival	[125]
ESCC	Downregulated	Tumor size, lymph node metastasis, poor prognosis	[126]
NSCLC	Downregulated	Survival rate	[130]
Glioma	Downregulated	Tumor grade	[60]
CRC	Downregulated	Lymph node metastasis, TNM staging, Overall survival	[129]
Glioblastoma	Downregulated	Survival	[76]
Breast cancer	Downregulated	Overall survival, Relapse-free survival, Distant metastasis-free survival, Disease-specific survival	[127]
Retinoblastoma	Downregulated	Survival	[128]
Glioma	Downregulated	Overall survival, Progression-free survival	[123]

Abbreviations: ESCC: esophageal squamous cell carcinoma; NSCLC: non-small cell lung carcinoma; CRC: colorectal cancer.

## Abbreviations

ABCG2: ATP binding cassette subfamily G member 2; AIFM2: apoptosis-inducing factor mitochondrion-associated 2; ALG9: alpha-1,2-mannosyltransferase; APAF-1: apoptotic protease activating factor-1; ATG3: autophagy related 3; ATG5: autophagy related 5; ATF6: activating transcription factor 6; ATG12: autophagy related 12; Bax: Bcl-2-associated X; Bcl-2: B-cell lymphoma 2; bFGF: basic fibroblast growth factor; Bmi1: B lymphoma Mo-MLV insertion region 1; BRI3: brain protein I3; BTG2: B-cell translocation gene; ceRNA: competitive endogenous RNA; CHOP: C/EBP-homologous protein; CLU: Clusterin; CNV: copy number variation; CRC: colorectal cancer; DDP: cisplatin; DNMT1: DNA methyltransferase 1; DRE: distal regulatory region; EGFR: epithelial growth factor receptor; EMT: epithelial-to-mesenchymal transition; EN-2: Engrailed-2; ER: endoplasmic reticulum; EZH2: enhancer of zeste homologue 2; FoxM1: forkhead box M1; FOXO1: forkhead box O1; GRP78: glucose-regulated protein 78; GSDMD: gasdermin D; GSK: glycogen synthase kinase; Gtl2: gene trap loci 2; HDAC1: histone deacetylase 1; HK2: hexokinase 2; HMGA2: high mobility group AT-hook 2; H3K27me3: trimethylation of histone H3 lysine 27; JARID2: Jumonji and AT-rich interaction domain containing 2; KPS: karnofsky performance score; LATS2: large tumor suppressor 2; LC3 II: microtubule-associated protein light chain 3 II; LDHA: lactate dehydrogenase A; LncRNAs: long non-coding RNAs; MCL-1: myeloid cell leukemia-1; MDM2: murine double minute 2; MDR1: multidrug resistance protein 1; MEG3: maternally expressed gene 3; miRNA: microRNA; MMP-2: matrix metalloproteinase 2; MMP-3: matrix metalloproteinase 3; MMP-9: matrix metalloproteinase 9; MREs: microRNA response elements; MRP1: multidrug resistance-associated protein-1; NF-κB: nuclear factor-κB; OTUD4: OTU deubiquitinase 4; OS: overall survival; PBLD: phenazine biosynthesis-like domain-containing; PCNA: proliferating cell nuclear antigen; PD-1: programmed death-1; PDCD4: programmed cell death 4; PERK: protein kinase R-like endoplasmic reticulum kinase; PGF: placental growth factor; PHLPP2: PH domain and leucine-rich repeat protein phosphatase 2; PKD1: protein kinase D1; PKM2: pyruvate kinase muscle isozyme M2; PRC2: polycomb repressive complex 2; PSAT1: phosphoserine aminotransferase 1; p-STAT3: phosphorylated signal transducer and activator of transcription 3; PTX: paclitaxel; PTEN: phosphatase and tensin homolog; Rac1: Rac family small GTPase 1; RASL11B: RAS like family 11 member B; RBMS3: RNA binding motif single stranded interacting protein 3; SDF-1: stromal cell-derived factor-1; SDH: succinate dehydrogenase; SOCS3: cytokine signaling 3; sORFs: short open reading frames; SOCS5: suppressor of cytokine signaling 5; SOCS6: suppressor of cytokine signaling 6; SOX7: SRY-box transcription factor 7; ST3Gal1: β-galactoside α-2,3-sialyltransferase 1; TGFBR1: transforming growth factor beta receptor 1; TGF-β1: transforming growth factor β1; RNF2: RING finger protein 2; TPM1: tropomyosin 1; TRAF3: TNF receptor-associated factor 3; VEGFA: vascular endothelial growth factor A; VEGFB: vascular endothelial growth factor B.
